# Treatment of peritoneal metastases from colorectal cancer

**DOI:** 10.1093/gastro/gov044

**Published:** 2015-09-30

**Authors:** Loreen März, Pompiliu Piso

**Affiliations:** Department of Surgery, St. John of God Hospital Regensburg, Regensburg, Germany

**Keywords:** colorectal cancer, peritoneal metastases, cytoreductive surgery, intraperitoneal chemotherapy

## Abstract

Peritoneal seedings of a colorectal tumor represent the second most frequent site of metastasis (after the liver). In the era of 5-fluorouracil (5-FU)-only chemotherapy, the prognosis was poor for colorectal cancer with peritoneal metastases. Within the last few years, new chemotherapeutic and targeted agents have improved the prognosis; however, the response to these treatments seems to be lower than that for liver metastases. The combination of cytoreductive surgery and hyperthermic intraperitoneal chemotherapy have further improved both disease-free survival and overall survival. Keeping this in mind, every patient presenting with peritoneal metastases from colorectal cancer should be evaluated and receive adequate treatment, if possible in the above-mentioned combination. This paper reviews recent advancements in the therapy of peritoneal carcinomatosis.

## Introduction

One unique feature of malignant tumor cells is their ability to spread to other organs via distribution through blood, lymph or peritoneal fluid. Once the primary tumor has spread, survival rates decrease rapidly.

Colorectal cancer is the third most common tumor worldwide; in 2035, 2.4 million patients will have developed a colorectal malignancy [1]. In colorectal cancer, the peritoneum is the second most-frequent site for metastases after the liver [2–4]. Maybe due to its poor prognosis, the incidence of peritoneal carcinomatosis has been widely overrated in previous studies. Whereas data-based estimates have shown the peritoneum as the sole site of metastatic disease in up to 25% of all cases, recent studies show that only 10% of patients have isolated peritoneal carcinomatosis. However, up to 20% may have peritoneal metastases with liver or other organ metastases [5]. Nevertheless, the occurrence of peritoneal carcinomatosis is associated with poor prognosis: With no treatment, median survival is six to nine months [2,3,6,7].

In order to treat peritoneal metastases effectively, various approaches have been made over the past decades. In this study, we will review the current treatment options for colorectal peritoneal metastatic disease.

## Systemic chemotherapy and targeted therapeutic agents

For a long time, peritoneal metastases have been regarded as a form of systemic distant metastatic disease and therefore the terminal stage of the disease. Only palliative systemic chemotherapy was used, and the few reported retrospective studies showed disappointing responses for chemotherapy with 5-fluorouracil (5-FU) and leucovorin with patients seldom surviving as long as eight months [2,6,8,9]. However, during the last decade, more effective cytotoxic chemotherapies and biological targeted therapies have been developed. Oxaliplatin, irinotecan, bevacizumab, cetuximab, panitumumab or lately ramucirumab and aflibercept have succeeded in improving the survival of patients with metastatic disease in clinical studies. However, as most of the studies had been conducted to demonstrate the effect of chemotherapy for all kinds of metastatic colon cancer, and because the majority of patients involved in these studies suffered from liver or lung metastases, the effect of systemic chemotherapy on peritoneal metastatic disease still remains unclear. Moreover, patients suffering from peritoneal carcinomatosis are sometimes excluded from studies because of their heavy tumor burden. Only a few studies have utilized systemic chemotherapy alone for patients with peritoneal carcinomatosis, whereas most studies included a multidisciplinary approach with surgery and systemic chemotherapy.

Two big phase 3 trials, N9741 and N9841, compared FOLFFOX chemotherapy-only treatment with irinotecan in both patients with and witout peritoneal carcinomatosis. Franko *et al*. showed a 30% reduced overall survival in patients with peritoneal carcinomatosis. The FOLFOX regime is superior to both IFL (irinotecan plus 5-FU, Saltz regime) and irinotecan plus oxaliplatin (IROX) with no relation to the presence of peritoneal carcinomatosis [5].

Recently, several studies have been conducted on combination therapies for metastasized colorectal carcinoma. A triple regime consisting of 5-FU, folic acid oxaliplatin and irinotecan (FOLFOXIRI) has been proven more effective than FOLFIRI as first-line therapy [10]. Adding EGFR-targeted therapy to systemic therapy has been investigated in two prospective trials. The BOND trial showed better results using cetuximab with irinotecan as compared with cetuximab without irinotecan in patients with a progression of disease after three months of irinotecan-based chemotherapy [11]. The randomized phase 3 CRYSTAL study found both a slightly prolonged progression- 35 free survival and overall survival in wild-type GTPase KRas (KRAS) when treated with FOLFIRI and cetuximab as compared with FOLFIRI alone. In mutant KRAS, progression-free survival was reduced under treatment with cetuximab [12,13]. As a randomized phase 3 trial, the FIRE-3 trial compared FOLFIRI plus cetuximab with FOLFIRI plus bevacizumab in patients with metastatic colorectal cancer with KRAS wild-type and showed a median progression-free survival of 10.0 and 10.3 months, respectively. Median overall survival was prolonged in the cetuximab group (28.7 to 25.0 months). This study shows remarkable prolongation of both progression-free and overall survival when chemotherapeutics and targeted therapeutic agents were used [14].

As for the use of systemic chemotherapy in combination with cytoreductive surgery (CRS) and intraperitoneal chemotherapy, the COMBATAC study (NCT01540344) is the first clinical study to investigate the effect of CRS and hyperthermic intraperitoneal chemotherapy (HIPEC) within an interdisciplinary treatment regime of pre- and post-operative systemic chemotherapy including cetuximab. The study has finished recruiting and is expected to publish its results soon [15]. The ongoing trials are presented in Table 1.

**Table 1.** Ongoing trials ([from ClinicalTrials.gov and clinicaltrialsregister.eu]

Trial Label	Trial Name (Short)	Trial Name	Summary
NCT01226394	ProphyloCHIP	Trial Comparing Simple Follow-up to Exploratory Laparotomy Plus “in Principle” (Hyperthermic Intraperitoneal Chemotherapy) HIPEC in Colorectal Patients	Multicenter randomized trial. Patients with a high risk of developing colorectal peritoneal carcinomatosis after resection of their primary. Six months systemic chemotherapy (currently FOLFOX-4). In case of recurrence: best known treatment. No recurrence: randomization to surveillance alone (control group) or exploratory laparotomy + HIPEC (experimental group). (Elias, Villejuif)
NCT02231086	COLOPEC	Adjuvant HIPEC in High Risk Colon Cancer	Multicenter randomized trial. Adjuvant HIPEC followed by adjuvant chemotherapy (CAPOX) (experimental group) or adjuvant systemic chemotherapy (control group). Diagnostic laparoscopy after 18 months. (Tanis, Amsterdam)
NCT01815359	ICARuS	Post-operative Intraperitoneal Chemotherapy (EPIC) and Hyperthermic Intraperitoneal Chemotherapy (HIPEC) After Optimal Cytoreductive Surgery (CRS) for Neoplasms of the Appendix, Colon or Rectum With Isolated Peritoneal Metastasis	Multicenter randomized trial. Early post-operative intraperitoneal chemotherapy (EPIC) *vs* hyperthermic intraperitoneal chemotherapy (HIPEC). (Nash, New York)
NCT01580410	–	Surgery and Oxaliplatin or Mitomycin C in Treating Patients With Tumors of the Appendix	Multicenter randomized trial. Oxaliplatin or Mitomycin C as HIPEC in patients with appendiceal tumors. (Levine, Winston-Salem)
EudraCT 2006-006175-20	PRODIGE 7	Essai de phase III évaluant la place de la ChimioHyperthermie IntraPéritonéale peropératoire (CHIP) après résection maximale d'une carcinose péritonéale d'origine colorectale associée à une chimiothérapie systémique.	Multicentrer randomized trial. Adjuvant chemotherapy + CRS ± HIPEC for peritoneal metastases from colon cancer. (Quenet, Montpelier)

## Cytoreductive surgery and intraperitoneal chemotherapy

**Figure 1.**
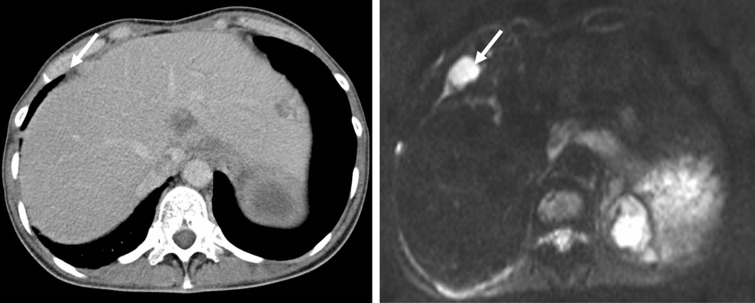
Peritoneal carcinomatosis from colorectal cancer in CT scan (left) and diffusion-weighted imaging (DWI), right. DWI shows lesions that are not visible in conventional CT scan (arrow).

For a long time, surgery in patients with peritoneal carcinomatosis arising from colon cancer was performed only to control the symptoms of the disease, such as bowel obstruction, bleeding and abdominal pain. Surgery was performed with palliative intent as diverting ileo- or colostomy or debulking of the abdominal tumor mass. This kind of surgery did not treat the underlying disease and was by no means meant to prolong life [16–19]. The concept of complete surgical cytoreduction has beendeveloped within the past 20 years [20–22]. The aim of CRS is to remove all macroscopically visible tumor load. Before the start of surgery, the Peritoneal Cancer Index (PCI) is evaluated. The PCI was first introduced by Sugarbaker in 1996. It ranges from 0 to 39 and assesses the extent of the disease by classifying the tumor size and the involvement of the parietal peritoneum and small bowel. The PCI can be determined prior to surgery by CT or MRI [23] (Figure 1). Another score used to describe the extent of peritoneal metastases is the Peritoneal Surface Disease Severity Score (PSDSS). This scoret classifies peritoneal metastases by measuring the symptoms of the patient, the PCI and the primary tumor histopathology [24]. Both PCI and PSDSS scores concur that a higher score, which means more extensive peritoneal metastatic disease, goes along with a poorer progression-free survival and a shortened overall survival.

**Figure 2.**
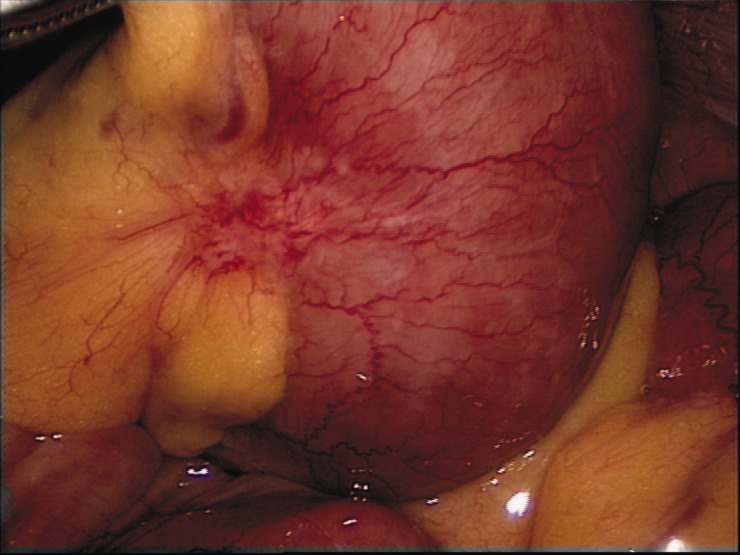
Small peritoneal carcinomatosis tumor nodules in the mesenterium of the small bowel.

The procedures performed with a CRS include multivisceral resection, frequently in combination with resection of the parietal peritoneum. Frequently performed surgery steps are omentectomy, colonic resection, resection of the parietal peritoneum of the pelvis and right upper quadrant followed by rectal resection and resection of parts of the small bowel (Figure 2). Resections of the liver and pancreas are limited to a few cases due to the increased morbidity and mortality of these procedures [25].

CRS can be a long and challenging procedure, therefore it is essential for patients undergoing this treatment to have generally good health. Major contraindications are severe diseases of the cardiovascular, respiratory and hepatorenal system, isolated or combined. Patients should not have evidence of tumor progress while on chemotherapy (Table 2) [26,27]. Extensive disease contraindicates CRS. Only 25% of patients diagnosed with peritoneal carcinomatosis of colorectal origin can be considered for a curative approach such as CRS [28]. Complete cytoreduction (CCR-0) is the key to a successful outcome and an essential prognostic factor. Studies have shown that patients with incomplete cytoreduction (CCR-1 or -2) and residual tumor more than 2.5mm do not gain any advantage in their survival and only have only about six months to live. The CCR score describes the size of the lesions left behind, ranging from CCR-0 (no visible tumor left) to CCR-2 (macroscopic tumor left) [28]. Vice versa, complete cytoreduction is associated with improved overall survival as well as disease-free survival as compared with patients who undergo only systemic chemotherapy [20,29].

**Table 2.** Selection criteria for cytoreductive surgery and hyperthermic intraperitoneal chemotherapy [from 23–27]


No extra-abdominal metastases	None to mild symptoms	No severe co-morbidities
Histology: Well/ moderately differentiated	Peritoneal Cancer Index (PCI) <20 (CT, PET-CT, Laparoscopy)	Patient‘s motivation
Not more than one bowel stenosis and no extensive small bowel disease (CT, PET-CT, Laparoscopy)	Not more than 3 peripheric resectable liver metastases	No biliary or ureteral obstruction (by tumor infiltration)
No tumor progress while on chemotherapy	No involvement of the gastrohepatic ligament >5cm (CT)	Acceptable quality of life achievable
CCR-0/-1 possible	EOCG 1 or 2	Informed consent

Complete tumor cytoreduction, combined with intraperitoneal use of chemotherapy was introduced into surgical practice as a treatment for metastatic colorectal cancer in 2007 [30]. The chemotherapeutics actually used for intraperitoneal chemotherapy in colorectal cancer are oxaliplatin in combination with intravenous 5-FU and leucovorin, mitomycin C, irinotecan, cisplatin and doxorubicin [31]. New chemotherapeutics such as humanized antibodies are currently being studied for their effect with intraperitoneal use. A phase 2 trial with catumaxomab as an intraperitoneally applied antibody in patients with peritoneal carcinomatosis from gastric cancer is currently running in France [32]. Nevertheless, bevacizumab, a monoclonal antibody that inhibits vascular endothelial growth factor-A has been tested in animal models without promising results [33].

The two most common application forms of intraperitoneal chemotherapy in colon cancer are HIPEC and early postoperative intraperitoneal chemotherapy (EPIC). HIPEC is usually performed directly after complete or almost complete surgical cytoreduction. When performing HIPEC, the abdominal cavity is perfused with a heated chemotherapeutic (generally 42° C) for 30 to 60 minutes. This perfusion takes place in the operating room with the patient still under anesthesia and can be conducted with the abdomen already closed or with an open abdomen (coliseum technique). Chemotherapeutics are applied heated because in vitro tests have demonstrated that certain drugs such as oxaliplatin, mitomycin C, doxorubicin, irinotecan and cisplatin increase their penetration depth, cytotoxic effect and therefore their antitumorous effect at a temperature of 42° C. HIPEC can be repeated if indicated. Given strict patient selection and careful indication, the morbidity and mortality of CRS with HIPEC are tolerable. In certified centers, the rate of grade 3 and 4 adverse events does not exceed 30%, while postoperative mortality is about 5% [27].

EPIC is performed postoperatively, within the first five days after surgery. Usually, the chemotherapeutic agents are applied unheated directly to the abdominal cavity through tubes placed during the preceding surgery. Several protocols exist with differences in the entrance point, the substance and the frequency of chemoperfusion. Morbidity and mortality are comparable with CRS with HIPEC [27].

From a pharmacological point of view, the intraperitoneal administration of chemotherapy is a very attractive approach. Higher drug concentrations can be used inside the peritoneal cavity without increasing systemic toxicity because of the peritoneal-plasma barrier. In animal models, intraperitoneal application of chemotherapeutics has successfully prevented the development of peritoneal carcinomatosis [34]. As to clinical studies, the benefit of intraperitoneal use for chemotherapeutics is not that obvious. Desolneux *et al*., in a prospective study with 103 patients undergoing CRS without HIPEC and postoperative systemic chemotherapy, demonstrated both increased overall survival and a disease-free survival similar to patients undergoing CRS and HIPEC [35]. A French study by Elias *et al.* was stopped for poor accrual; indeed the 2-year overall survival rate seemed to show no difference [36]. While there is no doubt that complete surgical cytoreduction is the key to prolonged disease-free survival and overall survival [37], intraperitoneal chemotherapy will have to produce evidence for its efficacy in the near future. As a prospective trial, the COLOPEC randomized multicentre trial (NCT02231086) is currently recruiting patients in order to investigate the effect of adjuvant intraperitoneal chemotherapy in patients with colon cancer and a high risk of developing peritoneal carcinomatosis [38].

## Conclusion

Since its humble beginnings with survival barely exceeding that of untreated patients, the therapy of peritoneal-metastasized colorectal carcinoma has made considerable progresses. The increased effectiveness of systemic chemotherapy in combination with targeted chemotherapy has improved the survival of patients with peritoneal metastases from colorectal cancer. Selected patients with localized peritoneal spread will benefit from additional surgical cytoreduction and regional heated chemotherapy with a further improval of their survival up to 48 months and a five-year probability of up to 50% [39]. Therefore, all patients with isolated peritoneal metastases should be evaluated in multidisciplinary teams in order to prove their suitability for a multimodality treatment strategy.

*Conflict of interest statement:* none declared.
